# A dosimetric comparison of different treatment plans of palliative spinal bone irradiation: analysis of dose coverage with respect to ICRU 50 report

**DOI:** 10.1186/1756-9966-28-2

**Published:** 2009-01-07

**Authors:** Fundagul Andic, Sule Baz Cifci, Yasemin Ors, Umar Niang, Ahmet Dirier, Mustafa Adli

**Affiliations:** 1 Department of Radiation Oncology, Faculty of Medicine, Gaziantep University, Gaziantep, Turkey

## Abstract

**Background:**

This study aimed to analyze three-dimensional (3D) dosimetric data of conventional two-dimensional (2D) palliative spinal bone irradiation using different reference points and treatment plans with respect to the International Commission on Radiation Units and Measurements (ICRU) Report 50.

**Methods:**

Forty-five simulation CT scans of 39 patients previously treated for thoraco-lumbar spinal bone metastases were used. Three different treatment plans were created: (1) single posterior field plans using the ICRU reference points (ICRUrps); (2) single posterior field plans using the International Bone Metastasis Consensus Working Party reference points (IBMCrps); (3) two opposed anterior-posterior (AP-PA) field plans using the ICRUrps. The intended dose range for planning target volume (PTV) was 90% to 110% of the prescribed dose for AP-PA field plans. Cumulative dose-volume histograms were generated for each plan, and minimum, maximum and mean doses to the PTV, medulla spinalis, esophagus and intestines were analyzed.

**Results:**

The mean percentages of minimum, maximum and mean PTV doses ± standard deviation were, respectively, 91 ± 1.3%, 108.8 ± 1.3% and 99.7 ± 1.3% in AP-PA field plans; 77.3 ± 2.6%, 122.2 ± 4.3% and 99.8 ± 2.6% in ICRUrp single field plans; and 83.7 ± 3.3%, 133.9 ± 7.1% and 108.8 ± 3.3% in IBMCrp single field plans. Minimum doses of both single field plans were significantly lower (p < 0.001) while maximum doses were significantly higher (p < 0.001) than AP-PA field plans. Minimum, maximum and mean doses were higher in IBMCrp single field plans than in ICRUrp single field plans (p < 0.001). The mean medulla spinalis doses were lower in AP-PA field plans than single posterior field plans (p < 0.001). Maximum doses for medulla spinalis were higher than 120% of the prescribed dose in 22 of 45 (49%) IBMCrp single field plans. Mean esophagus and intestinal doses were higher (p < 0.001) in AP-PA field plans than single field plans, however, less than 95% of the prescribed dose.

**Conclusion:**

In palliative spinal bone irradiation, 2D conventional single posterior field radiotherapy did not accomplish the ICRU Report 50 recommendations for PTV dose distribution, while the AP-PA field plans did achieve the intended dose ranges with a homogenous distribution and reasonable doses to the medulla spinalis, esophagus and intestines.

## Background

External beam radiotherapy is a well-recognized and effective modality in the palliation of symptomatic bone metastases and complication control [1]. Under- or overdosing the target volume and dose heterogeneity may not be major concerns, since many patients treated for palliative purposes have short survival. However, long term symptom control associated with bone involvement and normal tissue complications becomes more vital in cancer patients with long life-expectancy. Some breast and prostate cancer patients even with spinal cord compression may live for several years after radiotherapy.

Single posterior field or two opposed anterior-posterior fields (AP-PA) conventional two-dimensional (2D) radiotherapy planning without dose volume information is widely used for palliative spinal bone irradiation using the International Commission on Radiation Units and Measurements reference points (ICRUrps) and the International Bone Metastasis Consensus Working Party reference points (IBMCrps) [2,3].

To our knowledge, dosimetric assessment of conventional 2D palliative spinal bone irradiation using three-dimensional (3D) dose information has not been reported. This study aimed to analyze 3D dosimetric data of palliative spinal bone irradiation using different reference points and treatment plans with respect to the International Commission on Radiation Units and Measurements (ICRU) Report 50 [2].

## Methods

### CT simulation

Forty-five simulation CT scans of 39 patients previously treated for thoraco-lumbar spinal bone metastases were used for treatment planning. CT scanning was performed with a 6 detector helical CT (Brilliance, Philips Medical Systems, Netherlands) and with a 5-mm slice thickness.

### Volumes of interest

**Figure 1:**
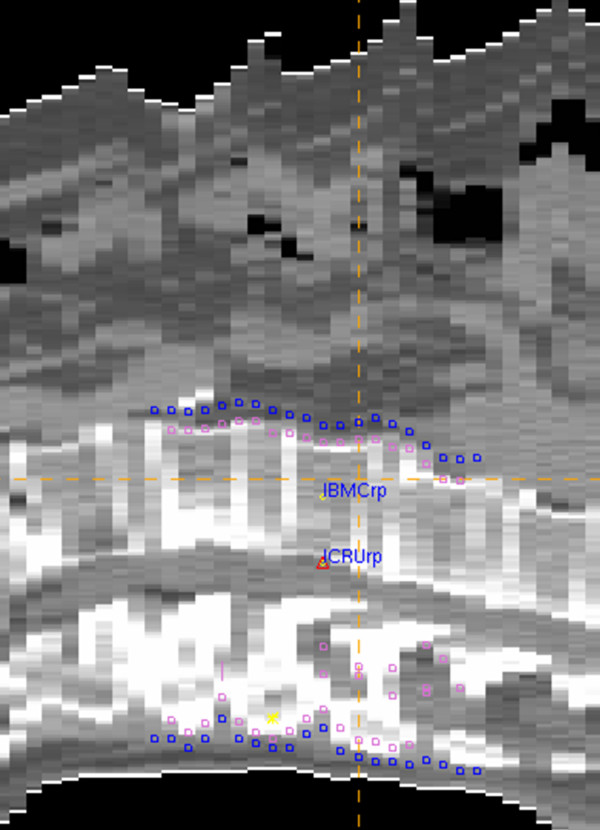
**Target volumes and reference points**. Clinical target volume (CTV), (pink line); planning target volume (PTV), (dark-blue line); ICRUrp, the International Commission on Radiation Units and Measurements reference point; IBMCrp, the International Bone Metastasis Consensus Working Party reference point.

Target volumes were contoured in corresponding CT slices (Figure 1). One vertebra above and below the involved vertebra(e) were included in the clinical target volume (CTV). However, the upper end-plate of the upper vertebra and the lower end-plate of the lower vertebra were not included in the CTV, to limit the distal and proximal borders of the treatment fields in the inter-vertebral space. To determine the planning target volume (PTV), 10 mm was added to CTV in lateral directions and 5 mm in anterior-posterior and superior-inferior directions. Treatment fields were determined by adding 7–10 mm to the PTV using multi-leaf collimators.

Portions of the esophagus located in thoracic radiotherapy fields, the intestines located in lumbar radiotherapy fields and the medulla spinalis in all fields were delineated as critical organs.

### Treatment planning

Precise PLAN^®^2.11 (Elekta, Crawley, UK) treatment planning system (TPS), which enables 3D conformal radiotherapy planning, was used for treatment plans. To calculate the dose distribution of the photon beam, the TPS uses an irregular field algorithm, for different depths and field sizes, based on data measures in a phantom. The algorithm takes into account the inhomogeneity of the patient's tissue and uses an integration scheme to evaluate the scatter component of the dose. The dose calculation grid is set to 2.5 mm.

Three different treatment plans were created (1) single posterior field treatment plans using ICRUrps; (2) single posterior field treatment plans using IBMCrps; and (3) two opposed anterior-posterior (AP-PA) field plans using ICRUrps.

**Figure 2:**
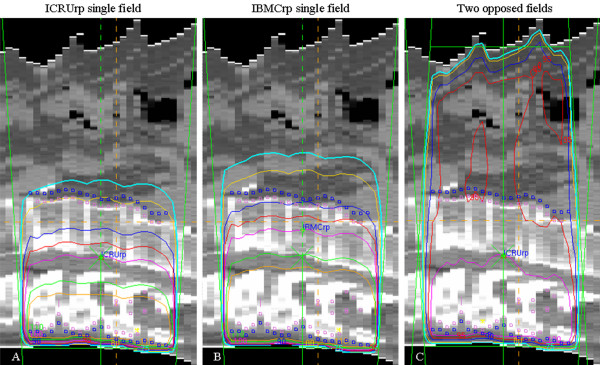
**Dose distributions in one case for ICRUrp single field plan (A), IBMCrp single field plan (B) and two opposed anterior-posterior field plan (C)**. ICRUrp, the International Commission on Radiation Units and Measurements reference point; IBMCrp, the International Bone Metastasis Consensus Working Party reference point. The isodose lines are shown as follows: 75% (blue), 80% (yellow), 90% (dark blue), 95% (red), 100 (pink), 110% (green), 115% (orange).

The ICRUrp was defined as the center of the PTV, the IBMCrp was defined as the mid-vertebral body point in the central plane, and the prescription dose was normalized to these points (Figure 1). Dose distributions of treatment plans in one case are shown in Figure 2.

The nominal prescribed dose was 2000 cGy in 5 fractions using 6-MV photons for posterior fields and 18-MV for anterior fields. In AP-PA field plans, beam weights were used as 1 and 1.5–2 in AP and PA fields, while assuring the intended dose range of 90% to 110% of the prescribed dose for the PTV. No dose constraint was used in single posterior field plans.

Cumulative dose-volume histograms (DVH) were generated for each plan, and minimum, maximum and mean doses to the PTV, medulla spinalis, esophagus and intestines were calculated for both single posterior fields and AP-PA field plans.

**Figure 3:**
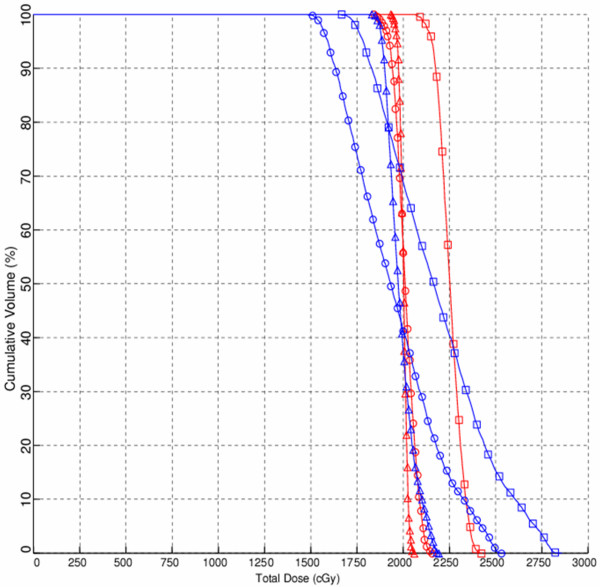
**Cumulative dose-volume histograms of one case for planning target volume (PTV) (dark-blue line) and medulla spinalis (red line) in single field plan using the International Commission on Radiation Units and Measurements reference point (circles), in single field plan using the International Bone Metastasis Consensus Working Party reference point (squares) and two opposed anterior-posterior field plan (triangles)**.

Cumulative dose-volume histograms of treatment plans in one case for PTV and medulla spinalis are shown in Figure 3.

### Statistical analysis

The mean, minimum and maximum dose levels were compared using the Paired-Samples T test for parametric data on the PTV and medulla spinalis and the Wilcoxon test for non-parametric data on the esophagus and intestines. P-values of less than 0.05 were considered statistically significant. Values are expressed as mean (range) ± standard deviation (SD).

## Results

Dose ranges of the PTVs for all plans are shown in Table 1. AP-PA field plans achieved the intended dose ranges and homogeneity for PTVs, unlike the single posterior field plans. Minimum doses of both single posterior field plans were significantly lower (p < 0.001) while maximum doses were significantly higher (p < 0.001) than AP-PA field plans. Minimum, maximum and mean doses were higher in IBMCrp single field plans with an increased dose heterogeneity than in ICRUrp single field plans (p < 0.001).

**Table 1:** The mean percentages of minimum, maximum and mean planning target volume (PTV) doses ± standard deviation for all plans

	Mean dose (range) % ± SD		
	
	Single field-ICRUrp	Single field-IBMCrp	Two opposed fields
Minimums	77.3 (72–81) ± 2.6	83.7 (74–89) ± 3.3	91 (90–95) ± 1.3
Maximums	122.2 (114–130) ± 4.3	133.9 (115–147) ± 7.1	108.8 (104–110) ± 1.3
Means	99.8 (94–107) ± 2.6	108.8 (95–116) ± 3.3	99.7(97–102) ± 1.3

ICRUrp, the International Commission on Radiation Units and Measurements reference point; IBMCrp, the International Bone Metastasis Consensus Working Party reference point; SD, standard deviation.

The mean depth of the PTV from skin surface in the central plane was 9.8 (7.4–13.5) ± 1.1 cm and the mean patient thickness was 22.1 (14.4–29.1) ± 3.7 cm. Only in two plans were the ICRUrps and IBMCrps located at the same sites, which were in the mid-vertebral body. Of 45 ICRUrps, 35 were located on the medulla spinalis behind the vertebral body and 8 were located in the posterior 1/3 of the vertebral body. None of the ICRUrps were located in the anterior half of the vertebral body or anterior to the vertebral body.

The mean dose, expressed as percentages of the prescribed dose, to the portion of the esophagus in the thoracic radiotherapy fields was 78.6% (70–85%) ± 4.1% in the ICRUrp single field plans, 84.6% (74–92%) ± 5.5% in the IBMCrp single field plans and 94.5% (87–99%) ± 3.1% in the AP-PA field plans. The mean dose to the intestines located in the lumbar radiotherapy fields was 66.2% (58–78%) ± 5.1% in the ICRUrp single field plans, 73.1% (64–88%) ± 6.2% in the IBMCrp single field plans and 90.8% (82–99%) ± 3.7% in the AP-PA fields plans. The mean doses to the esophagus and intestines were higher in the AP-PA field plans than in the single posterior field plans (p < 0.001).

Dose ranges to the medulla spinalis for all plans are shown in Table 2. The mean doses to the medulla spinalis were lower in the AP-PA field plans than in the single posterior field plans (p < 0.001).In all IBMCrp single field plans, maximum doses to the medulla spinalis were greater than 115% of the prescribed dose and in 22 of 45 (49%) plans the maximum doses were greater than 120% of the prescribed dose. In only 4 ICRUrp single field plans did the medulla spinalis receive a dose greater than 115% of the prescribed dose. In the AP-PA field plans, none of the doses to the medulla spinalis exceeded 106% of prescribed dose.

**Table 2:** The mean percentages of minimum, maximum and mean medulla spinalis doses ± standard deviation for all plans

	Mean dose (range) % ± SD		
	
	Single field-ICRUrp	Single field-IBMCrp	Two opposed fields
Minimums	94.2 (85–102) ± 3.0	103.4 (96–109) ± 3.3	96.2 (94–101) ± 1.5
Maximums	108.8 (101–118) ± 3.6	120.1 (115–129) ± 3.5	103.2 (101–106) ± 1.4
Means	102 (95–112) ± 3.1	112.7 (107–117) ± 2.3	100.3 (98–104) ± 1.3

ICRUrp, the International Commission on Radiation Units and Measurements reference point; IBMCrp, the International Bone Metastasis Consensus Working Party reference point; SD, standard deviation.

## Discussion

The results of the present study showed that neither IBMCrp nor the ICRUrp single posterior field plans accomplished the ICRU Report 50 recommendations for dose distribution, while the AP-PA field plans achieved the intended dose ranges and homogeneity.

The ICRU Report 50 recommends selecting a reference point that is clinically relevant and representative of the dose distribution throughout the PTV, where the dose can be accurately determined and where there is no large dose gradient [2]. The point located at the center or central part of the PTV generally fulfills these requirements and is recommended as the ICRU reference point (ICRUrp). While a homogeneous dose within 95% to 107% of the prescribed dose is recommended for the target volume, a variation of ± 10% from the prescribed dose is widely used in clinical practice and was used in the present study for AP-PA field plans [2].

Thoracic and lumbar spinal irradiation is performed either with a single posterior field or two opposed AP-PA fields [4]. The International Bone Metastasis Consensus Working Party recommends dose prescriptions to the mid-vertebral body for single-posterior fields and including at least one vertebral body above and below the involved vertebra(e) in the treatment volumes [3]. However, these recommendations are not supported with dosimetric data or treatment outcomes.

Radiotherapy planning and delivery, and dose distribution may affect treatment outcome by dose coverage and dose heterogeneity in the target volume. Although several studies investigated optimal radiotherapy fractionation, the dose-volume effect on radiotherapy outcome, in terms of pain relief and duration of response, has not been evaluated [5-13]. Furthermore, higher re-treatment rates have been reported in single-fraction palliative radiotherapy than in multifraction radiotherapy [12-14]. The relation between higher re-treatment rates and physician bias, primary site, pain severity and duration of symptoms has been evaluated, but the relation between high re-treatment rates and dose coverage has not been investigated. Studies investigating the relationship between radiotherapy technique and treatment outcome would provide important information, particularly for patients with long life-expectancies.

Dose heterogeneity may become vitally important in patients with long life expectancies. Minimum target volume doses as low as 70% of the prescribed dose may diminish treatment success, while maximum target volume doses reaching as high as 130% of the prescribed dose may cause serious normal-tissue side effects in such patients. In the present study, the mean minimum dose for PTV in the ICRUrp single field plans was 77.3% (72–81%) ± 2.6% of the prescribed dose, and the mean maximum dose for PTV in the IBMCrp single field plans was 133.9% (115–147%) ± 7.1% of the prescribed dose. When the medulla spinalis doses were assed, maximum doses were higher than 120% of the prescribed dose in 22 of 45 (49%) IBMCrp single field plans but lower than 106% of prescribed dose in all AP-PA field plans. When the dose distribution to the esophagus and intestines were evaluated, mean doses were higher in the AP-PA field plans than the single field plans, but less than 95% of the prescribed dose.

## Conclusion

In palliative spinal bone irradiation, 2D conventional single posterior field radiotherapy did not accomplish the ICRU Report 50 recommendations for PTV dose distribution, however, two opposed AP-PA field treatment plans did achieve the intended dose ranges with a homogenous dose distribution and reasonable doses to the medulla spinalis, esophagus and intestines.

In patients with long life-expectancies, care must be taken to obtain a homogenous dose distribution throughout the target volume and conformal treatment plans rather than single field treatment plans should be considered in these patients.

## Abbreviations

3D: three-dimensional; 2D: two-dimensional; ICRU: International Commission on Radiation Units and Measurements; ICRUrp: the International Commission on Radiation Units and Measurements reference point; IBMCrp: the International Bone Metastasis Consensus Working Party reference point; AP-PA: two opposed anterior-posterior fields; PTV: planning target volume; CTV: clinical target volume; TPS: treatment planning system; DVH: dose-volume histograms; SD: standard deviation.

## Competing interests

The authors declare that they have no competing interests.

## Authors' contributions

FA conceived of the study, coordinated the study, helped acquisition of data, performed the statistical analysis and draft the manuscript. SB has performed treatment plans, participated in acquisition of data and helped to draft the manuscript. YO has performed treatment plans, participated in acquisition of data and helped to draft the manuscript. UN has been helped acquisition of data and drafting the manuscript. AD has been helped acquisition and analysis of data and helped to draft the manuscript. MA have participated in the conception and design of the study and revising the manuscript critically for important intellectual content. All authors read and approved the final manuscript.
